# Inactivation of *Escherichia coli* Using Nanosecond Electric Fields and Nisin Nanoparticles: A Kinetics Study

**DOI:** 10.3389/fmicb.2018.03006

**Published:** 2018-12-12

**Authors:** Vitalij Novickij, Auksė Zinkevičienė, Ramunė Stanevičienė, Rūta Gruškienė, Elena Servienė, Iglė Vepštaitė-Monstavičė, Tatjana Krivorotova, Eglė Lastauskienė, Jolanta Sereikaitė, Irutė Girkontaitė, Jurij Novickij

**Affiliations:** ^1^Institute of High Magnetic Fields, Vilnius Gediminas Technical University, Vilnius, Lithuania; ^2^Department of Immunology, State Research Institute Centre for Innovative Medicine, Vilnius, Lithuania; ^3^Laboratory of Genetics, Nature Research Centre, Vilnius, Lithuania; ^4^Department of Chemistry and Bioengineering, Vilnius Gediminas Technical University, Vilnius, Lithuania; ^5^Institute of Chemistry, Vilnius University, Vilnius, Lithuania; ^6^Institute of Biosciences, Life Sciences Centre, Vilnius University, Vilnius, Lithuania; ^7^Institute for Telecommunications, Vilnius Gediminas Technical University, Vilnius, Lithuania

**Keywords:** antimicrobial resistance, bacteria inactivation, *E. coli*, electroporation, nisin

## Abstract

Nisin is a recognized bacteriocin widely used in food processing, however, being ineffective against gram-negative bacteria and in complex food systems. As a result, the research of methods that have cell wall–permeabilizing activity is required. In this study, electroporation to trigger sensitization of gram-negative bacteria to nisin-loaded pectin nanoparticles was used. As a model microorganism, bioluminescent strain of *E. coli* was introduced. Inactivation kinetics using nanosecond pulsed electric fields (PEFs) and nisin nanoparticles have been studied in a broad range (100–900 ns, 10–30 kV/cm) of pulse parameters. As a reference, the microsecond range protocols (100 μs × 8) have been applied. It was determined that the 20–30 kV/cm electric field with pulse duration ranging from 500 to 900 ns was sufficient to cause significant permeabilization of *E. coli* to trigger a synergistic response with the nisin treatment. The kinetics of the inactivation was studied with a time resolution of 2.5 min, which provided experimental evidence that the efficacy of nisin-based treatment can be effectively controlled in time using PEF. The results and the proposed methodology for rapid detection of bacteria inactivation rate based on bioluminescence may be useful in the development and optimization of protocols for PEF-based treatments.

## Introduction

The clinical incidence of drug-resistant microorganisms has increased dramatically nowadays, which influenced a growing need for the development of new antimicrobial methods against major foodborne pathogens (McCrackin et al., 2016; Colavecchio et al., 2017). According to World Health Organization (WHO), foodborne illnesses affect more than 600 million people and cause 420,000 deaths annually (Wimley and Hristova, 2011; WHO, 2015; Colavecchio et al., 2017). The potential of antimicrobial peptides in this area is high; however, limitations for safety and biocompatibility apply in food industry, restricting the array of applicable peptides (Gharsallaoui et al., 2016). Currently, nisin is one of the few bacteriocins approved as a food preservative, which influences its common use in food technology and biomedical applications (Prudêncio et al., 2015; Shin et al., 2016; Li et al., 2018).

Nisin is produced by certain strains of *Lactococcus* spp. and is effective against broad spectrum of gram-positive bacteria (Gharsallaoui et al., 2016; Rao et al., 2016; Campion et al., 2017). It incorporates itself in the cell membrane by binding to essential precursors for cell wall biosynthesis, which ultimately leads to the formation of pores, loss of solutes in bacteria, and subsequent cell death (Wiedemann et al., 2004; Novickij et al., 2018b). However, nisin application against gram-negative bacteria is limited due to nonpermeability of outer membrane (Helander and Mattila-Sandholm, 2000).

As a result, the research of methods, which have cell wall–permeabilizing activity allowing nisin to be exposed to the inner membrane, is constantly performed (Murdock et al., 2007; Liu et al., 2015; Rao et al., 2016). Another limitation of nisin is associated with low antimicrobial activity in complex food systems, as it interacts with proteins and lipids or is inactivated by enzymatic degradation (Chopra et al., 2014; Wu et al., 2016). To address this problem, various types of nisin nanoparticles have been developed, which allow to improve the stability and release the bioactivity with reduced negative effects of the environment (Prombutara et al., 2012; Wu et al., 2016; Vukomanović et al., 2017). Electroporation could be the perfect candidate to trigger a synergistic effect with nisin nanoparticles and enable an effective food processing irrespective on the type of the bacterial contaminant.

Electroporation is a pulsed electric field (PEF)–induced phenomenon of increased membrane permeabilization, which can be precisely controlled by the parameters of the electrical pulses (Tsong, 1991; Rems and Miklavčič, 2016). It is a predominantly nonthermal method, which already has broad application in the food industry and biotechnology for extraction of proteins, sterilization, and biomass processing (Mahnič-Kalamiza et al., 2014, 2015; Golberg et al., 2016). In previous pilot study, it was shown that electroporation when combined with nisin nanoparticles triggers sensitization of gram-negative bacteria (Novickij et al., 2016c). It is hard for microorganisms to acquire PEF resistance due to the physical nature of the method; therefore, PEF treatment is universal (Pol et al., 2000; Del Pozo et al., 2008). Taking into account the availability and spread of PEF-based food processing technologies (Sitzmann et al., 2016; Chemat et al., 2017), the incorporation of nisin nanoparticles to the available technological process is straightforward. However, the major limiting factors are the lack of knowledge on the bioactivity of nisin nanoparticles and the kinetics of inactivation, including the unavailability of parametrical analysis of the synergistic responses, triggered by the combined treatment.

In this work, a focus was placed on the inactivation of *E. coli* (as a cell model) using PEF and nisin nanoparticles in a broad range of PEF parameters. This is, to our knowledge, the first study reporting on bacterial inactivation kinetics using the aforementioned techniques. The obtained bacterial inactivation kinetics was determined by a proposed bioluminescence assay, features high-time resolution (i.e., less than 3 min), and may be useful in the development of resource-effective PEF protocols or technological steps for PEF/nisin food processing when used in real food applications.

## Materials and Methods

### Electroporation Setup

**Figure 1:**
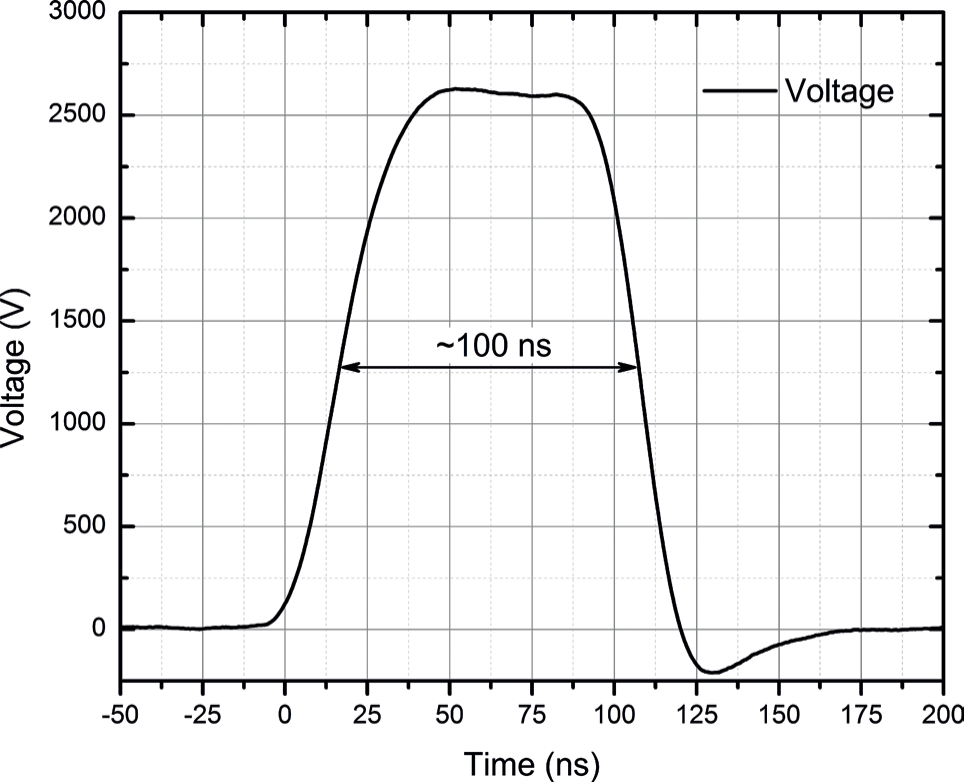
The waveform of the shortest applied pulse (100 ns). Acquired using DPO4034 digital oscilloscope (Tektronix, Beaverton, USA), postprocessed in Origin 8.5 (Origin Lab, Northampton, USA).

Up to 3 kV, 100 ns–1 ms square-wave high-voltage pulse generator was used for electroporation (Novickij et al., 2016b). The setup generated pulsed electric field (0–30 kV/cm) using several pulsing protocols: 1) 8 × 100 μs, 1 kHz and 2) 500 × 100–900 ns, 1 kHz. The pulses were generated in a commercially available 1-mm gap electroporation cuvette (Bio-Rad, Hercules, USA). The waveform of the shortest applied pulse (100 ns) is shown in Figure 1.

The waveform features low impact of transient processes with a negligible (<10%) reverse voltage, which is occurring due to specific structure (controlled crowbar circuit) of the applied pulse generator. The study covers both microsecond (conventional) and nanosecond range protocols due to the increasing interest in ultrashort pulsing (Guionet et al., 2014). The temperature rise due to ohmic heating was below 4°C during the highest energy density PEF (30 kV/cm, 8 × 100 μs, 15.6 kJ/L) and even lower during other protocols.

### Preparation of Bioluminescent *E. coli*

As a cell model, *E. coli* DH5α was used, which is a widely adopted model organism for laboratory studies and industrial application of PEF and food technology (Žgalin et al., 2012; Pan et al., 2014). The bacterial strain was transformed by electroporation with plasmid pAKlux2 (kindly provided by Attila Karsi, Addgene plasmid #14080) (Karsi and Lawrence, 2007). This plasmid contains the luxCDABE operon encoding luciferase (luxAB) and the enzymes that produce its substrate (lux CDE) genes. Therefore, the live (but not dead) bacteria are bioluminescent. The bacteria were grown in Luria-Bertani (LB) medium (2% tryptone, 2% yeast extract, and 1% NaCl) for 16–18 h with continuous shaking at 37°C. For exponential growth, 1 ml of overnight cultures were transferred to fresh LB medium (start optical density at 600 nm is about 0.1) and incubated at 37°C for additional 4 h (final OD600 is 1). Later, the cells (1 × 10^9^ cells/sample) were collected by centrifugation at 6,000 *g* for 5 min, washed three times with 1 M sorbitol, and resuspended in 1 M sorbitol at final concentration of 10^9^.

### Preparation of Nisin Nanoparticles

Nisin (NisinZ^TM^ P) was purchased from Handary S.A. (Brussels, Belgium). Pectic acid (PecA, Mw 30,000) was purchased from Serva (Heidelberg, Germany). Sorbitol was obtained from LEK D.D. (Slovenia). All reagents were used without additional purification. Nisin-loaded PecA nanoparticles were prepared as described previously (Krivorotova et al., 2016) with slight modifications. Briefly, prior to use, PecA was dissolved in distilled water at the concentration of 1 mg/ml by adjusting the pH of solution to 6.0 with 0.1 M NaOH. The stock solution of nisin in water at the concentration of 2 mg/ml was filtered through 0.2-μm pore size filters, and the pH of solution was adjusted to 6.0. For the formation of nanoparticles, a volume of nisin solution at the concentration of 1 mg/ml was added dropwise to the PecA solution under constant stirring at room temperature. Prior to the addition of nisin, PecA solution was mixed with 5 M sorbitol solution and diluted with water to obtain, finally, a nisin-pectin mixture at the PecA and nisin concentration of 0.4 mg/ml and at a sorbitol concentration of 1 M. Finally, the pH of the solution was adjusted to the value of 6.0. Determination of nisin loading efficiency and physicochemical characteristics of nanoparticles was performed according to Krivorotova et al. (2016). For the selected *E. coli* strain at pH 6.0, soluble nisin had a MIC value of 29.8 μM. At the same time, stabilized pectin encapsulated nisin nanoparticles had a MIC of 14.9 μM.

### Permeabilization Assay

For detection of cell permeabilization due to PEF, the propidium iodide (PI) (Thermo Fisher Scientific Inc., USA) fluorescent dye was used. Before electroporation, the 63 μl cell suspension was mixed with 7 μl of 300 μM PI to obtain 30 μM final dye concentration. For the experiments, 60 μl of the resultant suspension was transferred to 1-mm gap electroporation cuvette (Bio-Rad, Hercules, USA), and the pulses were applied. After electroporation, the cells were instantly transferred to 1.5 ml tubes (Eppendorf, Hamburg, Germany) and incubated for additional 10 min at room temperature followed by flow cytometric analysis (Amnis, Seattle, USA). PI fluorescence was evaluated using bandpass filter of 610–630 nm. The fluorescent cells (PI permeable) were gated as permeabilized in accordance with typical gating strategies used in electroporation studies (Michie et al., 2000; Novickij et al., 2016a, 2018a). A shift of fluorescence spectra and the cells in the defined gate (which was defined based on the untreated control) have been interpreted as fluorescence positive (permeabilized), while the cells outside the gate have been interpreted as nonfluorescent (nonpermeabilized). The percentage of the PI fluorescent cells in the untreated control did not exceed 10%, which was also confirmed by fluorescence imaging (Amnis, Seattle, USA).

### Viability Assay

After the treatment by nisin and/or PEF, the samples were incubated at room temperature (20°C) for 2 h; serial dilutions performed in sterile 0.9% NaCl; and 50 μl of each solution was spread onto LB-agar plates with following incubation overnight at 37°C. After incubation, colonies were counted as colony-forming units (CFUs), and then the mean value of CFU/ml was calculated.

### Spectrophotometry and Analysis of Kinetics

The bioluminescence of bacteria was evaluated using a Synergy 2 microplate reader and Gen5^TM^ software (BioTek, USA). The luminescent signal was measured kinetically for a period of 6 h with 2.5 min intervals, and relative luminescence units (RLUs) were detected for each sample. During evaluation, the cells were incubated in 1 M sorbitol at room temperature.

### Statistical Analysis

One-way analysis of variance (ANOVA; *p* < 0.05) was used to compare results. If ANOVA indicated a statistically significant result (*p* < 0.05), Tukey HSD multiple comparison test for evaluation of the difference was used. These data were further analyzed in OriginPro software (OriginLab, Northampton, MA, USA). All experiments were performed at least in three repetitions, and the results were expressed as means ± standard deviations.

## Results

**Figure 2:**
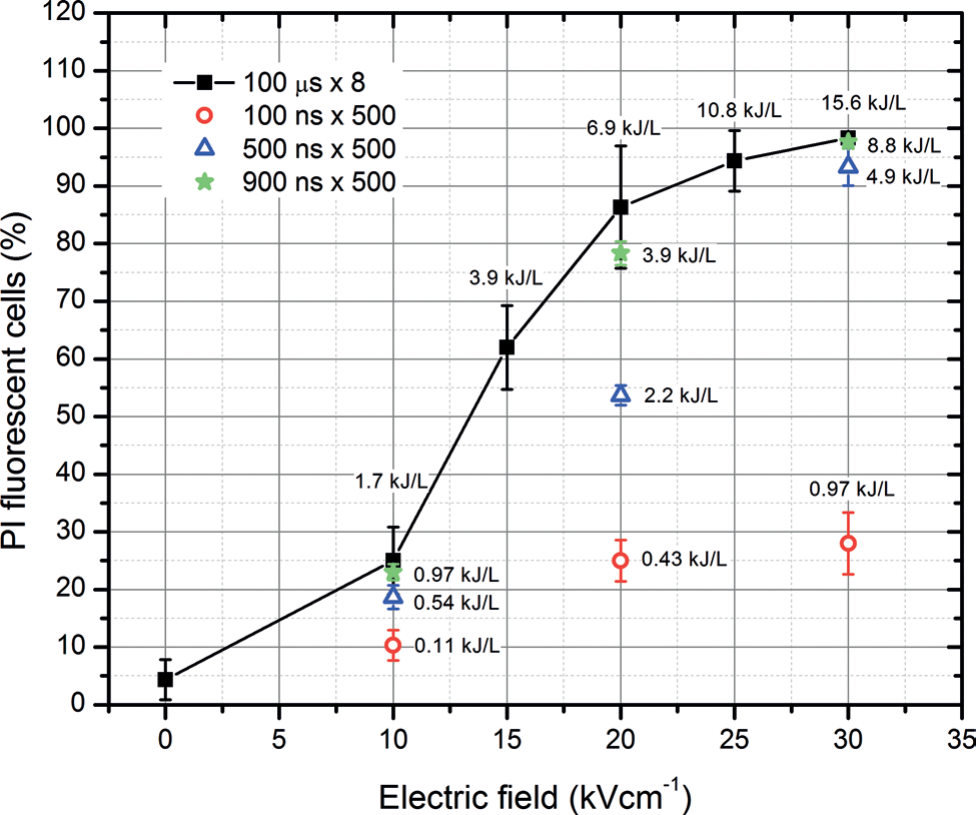
The dependence of cell permeabilization rate (propidium iodide fluorescent cells) on the applied electrical pulse parameters.

First, the dependence of cell permeabilization on the applied PEF treatment parameters was evaluated. The nanosecond range protocols have been compared with the conventional microsecond range electroporation (higher energy density of the pulse burst). The results are summarized in Figure 2.

As shown in Figure 2, the percentage of permeabilized bacterial cells (PI permeable) depends on the amplitude of PEF and the energy density of the burst. Nevertheless, the 900 ns × 500 and 100 μs × 8 protocols showed that a saturation exists, when the permeabilization efficacy is no longer incremental with an increase in pulse duration. As expected, the 500 and 100 ns protocols showed a dose-dependent response and featured a weaker permeabilization when compared to higher energy pulses (*p* < 0.05). Based on these results, it was presumed that the best synergy with nisin nanoparticles is expected with the 100 μs and 900 ns protocols. It should be noted that nanosecond range pulses (900 ns) allowed to trigger higher permeabilization when compared to microsecond range pulses of equivalent energy.

**Figure 3:**
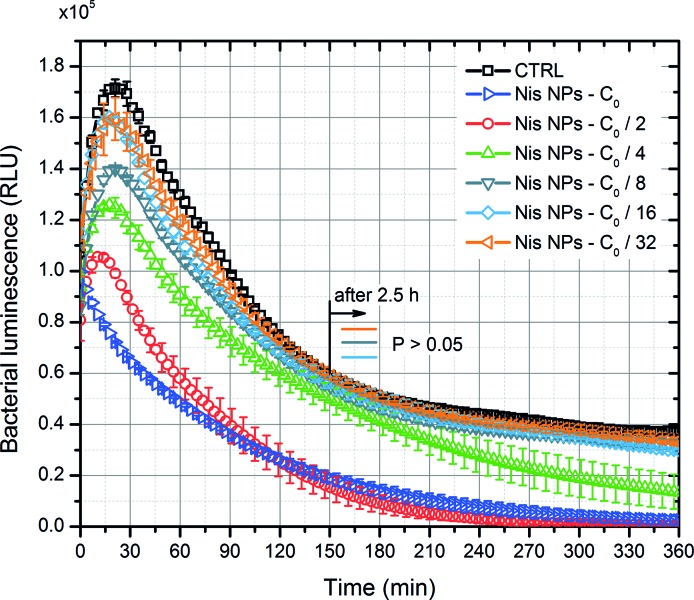
The dependence of bacteria luminescence on the concentration of nisin nanoparticles (Nis NPs). CTRL—control without nisin nanoparticles. C_0_ corresponds to highest concentration of 0.2 mg/ml.

The efficacy of nisin nanoparticles (Nis NPs) was further evaluated in the study. The initial concentration C_0_ of 0.2 mg/ml was diluted in incremental steps, and the resultant luminescence (corresponding to viability) was investigated during 6 h with a time step of 2.5 min. The results are summarized in Figure 3. The rise of luminescence during the first 30 min of the treatment was due to the exponential growing phase of bacteria. However, the luminescence intensity decreased with an increase in the Nis NPs concentration. The best efficacy was achieved, when the 0.1–0.2 mg/ml Nis NPs were used. The 8- to 32-fold diluted samples showed no statistically significant difference vs. untreated control; however, the same concentration-dependent pattern was apparent (*p* > 0.05).

For further experiments, the C_0_ concentration of Nis NPs was used, and the C_0_ curve (Refer to Figure 3) was taken as a reference of nisin-only treatment.

**Figure 4:**
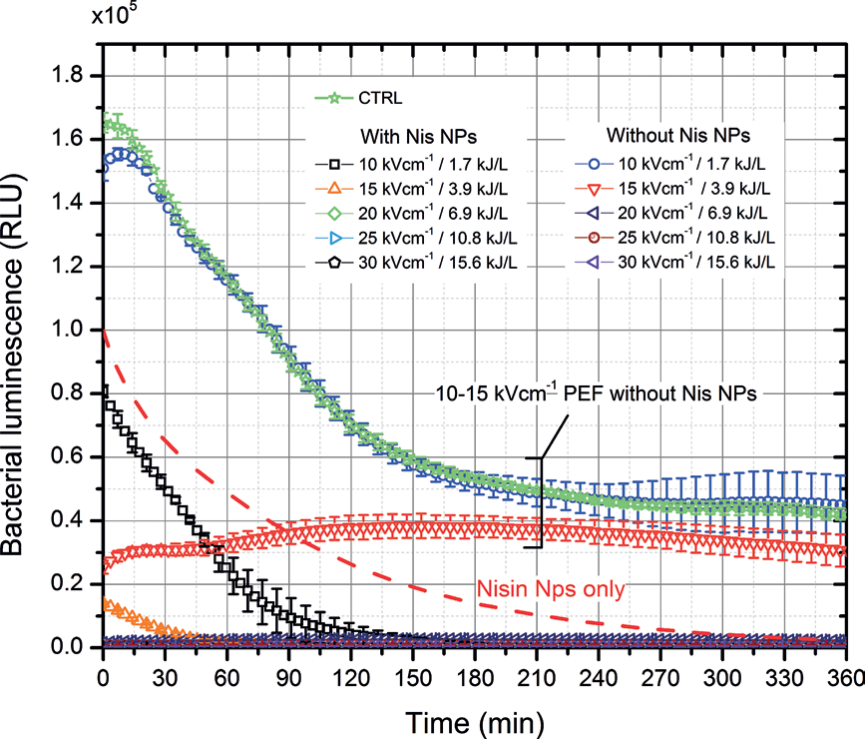
The dependence of bacteria luminescence on the applied combined pulsed electric field (100 μs × 8) and nisin nanoparticles (0.2 mg/ml) treatment.

The combination of two treatment methods was further investigated. The 0–30 kV/cm 100 μs × 8 PEF bursts with and without Nis NPs were applied, and the kinetics of luminescence decrease using the same methodology as described above was studied. The results are summarized in Figure 4. As shown in Figure 4, PEF itself allowed to reduce the bacterial luminescence when amplitudes higher than 20 kV/cm were used (predominantly irreversible electroporation is triggered). The combination of PEF with Nis NPs showed even higher luminescence decrease rates, and a clear synergistic response (Chou and Talalay, 1984) was apparent. Even 1.7 kJ/L, 10 kV/cm PEF (reversible electroporation) combined with Nis NPs resulted in more than 1 log reduction of RLU after 2 h of incubation. Lastly, it was determined that irreversible electroporation triggers instant reduction of cell bioluminescence—the highest loss in luminescence was detected instantly after the treatment.

**Figure 5:**
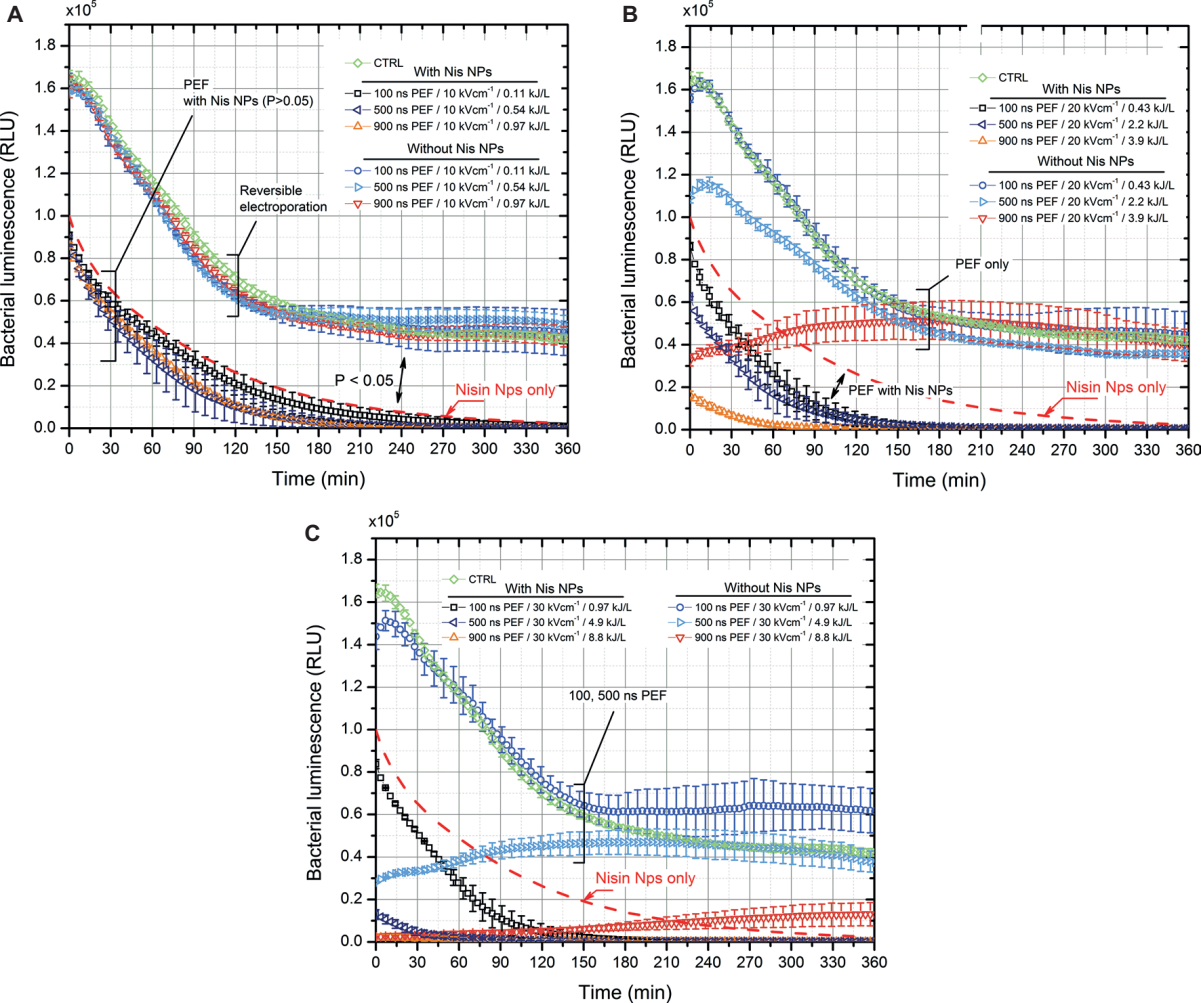
The dependence of bacteria luminescence on the applied combined pulsed electric field (100–900 ns × 500) and nisin nanoparticles (0.2 mg/ml) treatment, where **(A)** 10 kV/cm protocols; **(B)** 20 kV/cm protocols; **(C)** 30 kV/cm protocols; CTRL–corresponds to untreated control without nisin nanoparticles; dashed line is used as a reference to highlight the efficacy of nisin nanoparticles only treatment.

The impact of nanosecond range pulses was further evaluated in the study following the same methodology. The results are summarized in Figure 5. As shown in Figure 5A, the 10 kV/cm protocol, irrespectively from the duration of applied pulse, showed only a barely detectable additive effect with Nis NPs. The response was comparable to nisin-only treatment (dashed line). However, with an increase in PEF amplitude and energy density of the burst, the phenomenon of bacteria sensitization to nisin NPs was detected. The 20 kV/cm protocol triggered an additive effect with Nis NPs, and as expected, a dose-dependent response was apparent (Figure 5B). The PEF-only procedure featured a significant decrease in bacteria luminescence only after the 900 ns × 500 pulsing; however, after 2 h, the result was already comparable to control. Finally, the 30 kV/cm protocol was the most effective (Figure 5C). All the applied pulses (100–900 ns) resulted in luminescence decrease, and the additive effect with Nis NPs was detectable (*p* < 0.05).

**Figure 6:**
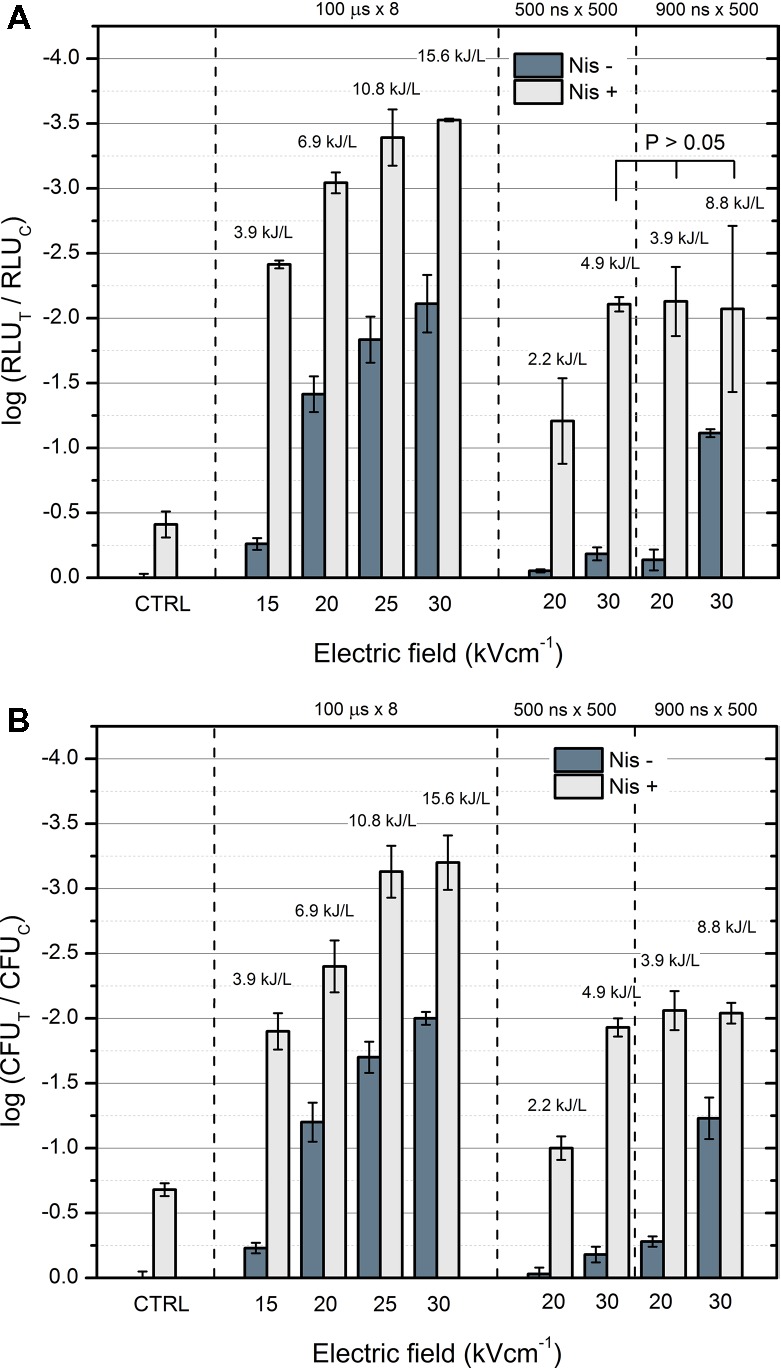
Comparison of the bacteria luminescence and viability assay under different treatment parameters after 2 h incubation with nisin nanoparticles, where **(A)** bioluminescence assay; **(B)** viability assay based on CFU count. “Nis −” corresponds to samples without nisin nanoparticles, and “Nis +” corresponds to samples with 0.2 mg/ml of nisin nanoparticles, RLU_T_ and RLU_C_ and CFU_T_ and CFU_C_ correspond to treated and control samples, respectively.

The experiment was further focused on the pulse parameters, which showed at least 1 log of RLU reduction after 2 h of incubation with Nis NPs. The bacteria bioluminescence assay was compared to viability assay (CFU count) to confirm the adequacy of the luminescence model for rapid detection of viability. The results are summarized in Figure 6. The decrease rates of the luminescence after treatment (Figure 6A) and the decrease rates of viability (Figure 6B) are in good agreement. The highest deviation between two methodologies was lower than 20%. Microsecond pulses resulted in higher inactivation of *E. coli* when compared to nanosecond range procedures due to higher energy density of the burst. Nanosecond-range protocols triggered predominantly reversible electroporation; however, high permeabilization rate of bacteria was sufficient to cause a synergistic response with nisin NPs. A saturation of the effect was reached in the range of 2 ± 0.2 log of CFU reduction for nanosecond pulses, while microsecond range protocols allowed triggering more than 3 log of CFU reduction.

## Discussion

Nisin is a recognized bacteriocin, which is widely used in food processing, however, commonly being ineffective against gram-negative bacteria and in complex food systems. As a result, various encapsulation methods are developed to overcome these limitations, and combinations with other methodologies (such as treatment by PEF) have been constantly researched (Mirhosseini and Afzali, 2016). It has been reported that PEF can be used to improve the efficacy of nisin (Smith et al., 2002; Saulis, 2010; Novickij et al., 2016c); however, the kinetics of inactivation are unknown, and the parametrical analysis has never been performed in the nanosecond range (Pol et al., 2000; Martín-Belloso and Sobrino-López, 2011). Therefore, in this study, the first report on the bacteria bioluminescence kinetics (corresponding to inactivation kinetics) (6 h, 2.5 min resolution) of gram-negative bacteria using Nis NPs in combination with nanosecond PEF was presented.

The nisin-loaded pectin nanoparticles (Krivorotova et al., 2016), as expected, showed limited efficacy against *E. coli* (Figure 6). This result is consistent since the antimicrobial effect of nisin is caused by its interaction with phospholipid components of the cytoplasmic membrane—the protective outer membrane, surrounding the cytoplasmic membrane, and peptidoglycan layer in gram-negative cells limits nisin permeability (Blake et al., 2011; Mirhosseini and Afzali, 2016). The lethality of the nisin-only treatment can be slightly improved by an increase in the incubation time (refer to Figure 3); however, in order to achieve practical applicability, additional stress (i.e., PEF or thermal influence) is required.

At the same time, PEF-only treatment (in the microsecond range) was effective by itself. A dose-dependent response was determined, which is in agreement with established electroporation theory (Pucihar et al., 2011). The inactivation mechanism using PEF is presumably straightforward and is associated with the physical damage (permeabilization) that is caused to the bacterial cells. The integrity and morphology of bacteria are sustained by the cell wall serving as a barrier against the environment. The high-intensity PEF induces disorganization with morphological, functional, and mechanical alterations of the cell wall (Pillet et al., 2016). Based on the acquired experimental data, the PEF-induced effect was instant and nonthermal. However, in this work, 1 M sorbitol was used as buffer, which ensured high impedance of the load (6–7 kΩ) and negligible thermal influence (< 4°C). Application of irreversible electroporation for food sterilization is often associated with an increase in temperature (Mahnič-Kalamiza et al., 2014) when high conductivity suspensions are processed. Mild increases of temperatures (either deliberate or due to ohmic heating) improve the antimicrobial efficacy of PEF; however, thermal influence may negatively affect the quality of food. In this study, low-energy density nanosecond pulse bursts were used (0.1–9 kJ/L) to sustain a nonthermal effect, however, sufficient to cause different levels of cell permeabilization with predominantly reversible permeabilization.

It was determined that the 30 kV/cm PEF with pulse duration ranging from 500 to 900 ns (4.9 and 8.8 kJ/L, respectively) was sufficient to cause high permeabilization of *E. coli* and trigger a synergistic response with nisin-loaded nanoparticles. Also, experimental evidence that the kinetics of the nisin-based inactivation (determined by the proposed bioluminescence assay) can be significantly increased by lower intensity (20 kV/cm, 0.43–3.9 kJ/L) PEF (Figure 5) was provided. The combined treatment allowed acquiring an improvement in inactivation efficacy (confirmed both by CFU count and bioluminescence data) up to several log of CFU reduction; however, a saturation of the efficacy was reached. The phenomenon is presumably associated with insufficient electrophoretic force, which is induced during nanosecond range pulses (Pakhomov et al., 2015). As a result, the intracellular electrotransfer of nisin is limited. In order to improve the efficacy, a further increase in the PEF amplitude and/or pulse duration is required.

Our data indicate that nanosecond pulses have a potential for inactivation of bacteria when used in combination with other treatments leading to a synergistic effect, which is in agreement with other studies (Guionet et al., 2015). Also, nanosecond range PEF methodologies are advantageous since the influence of electrochemical reactions (Chafai et al., 2015) can be reduced, which potentially may improve the durability of PEF applicators and overall quality of food if used in food processing. Lastly, due to the displacement currents, the interactions with subcellular structures are possible, and a more uniform permeabilization can be reached (Schoenbach et al., 2007; Joshi and Schoenbach, 2010).

In conclusion, the proposed bioluminescence assay may be successfully used for the assessment of the competence of different antimicrobial compounds and methods against *E. coli*. However, the study was limited to a single strain; therefore, future works should involve parametric analysis of PEF effects on multiple strains. Also, the inactivation rates may be different when the methods are applied in large batches or on bacteria in stationary growing phase. Nevertheless, we have shown a concept how encapsulation of nisin and application of nonthermal PEF allow achieving comparable efficiency even in ambient temperatures. Therefore, there is a high margin for efficiency improvement if the methodologies are used in mild thermal systems or with higher energy density of the pulses. The results are expected to be useful for the development of time-efficient and economically optimized PEF treatment protocols.

## Author Contributions

VN supervised the work. RG, TK, and JS developed and produced the nanoparticles and performed the characterization. VN, AZ, EL, and IG performed the spectrophotometry and flow cytometry, processed, and analyzed the results. ES supervised the microbiological experiments. RS and IV-M conducted the microbiological experiments, processed, and analyzed the results. VN and JN developed the pulsed power systems and applicators. VN, ES, JS, and JN interpreted the results. VN, ES, JS, JN, and AZ wrote the manuscript. All authors reviewed and approved the final manuscript.

### Conflict of Interest Statement

The authors declare that the research was conducted in the absence of any commercial or financial relationships that could be construed as a potential conflict of interest.
